# Supplementary material for: Analysis of the health economics portfolio funded by the National Institutes of Health in response to published guidance
Source: PLoS One. 2024 Feb 14;19(2):e0284235. doi: 10.1371/journal.pone.0284235 (PMC10866517; doi:10.1371/journal.pone.0284235)
Supplement: S1 Table — Compiled from several authoritative sources and used to build machine learning algorithm. (PDF) [file pone.0284235.s001.pdf]

S1 Table

Access to Health Care
Acquisition cost
Allocative Efficiency
Attitude to Health
Attributable fraction
Average Cost
Benefit-cost analysis
Benefit-cost ratio
budget impact analysis
Capital cost
Capitation
Clinical Effectiveness
Comorbidity
Consumer Behavior
Consumer Expenditure Survey
Consumer Price Index
Cost Allocation
Cost Analysis
Cost Comparison
Cost consequences analysis
Cost Control
Cost Description
Cost effectiveness analysis
Cost minimization analysis
Cost of Illness
Cost of illness analysis
Cost of Illness Study
Cost Outcome Description
Cost Sharing
Cost-benefit Analysis
Cost-effectiveness
Cost-minimization Analysis
Costs and Cost Analysis
Cost-utility Analysis
Decision-analysis
Decision-Making
Deductible
Direct benefits

Direct Service Costs
Disability adjusted life years
Discount rate
Drug Approval
Drug Costs
Drug Formulary
Drug Utilization
Economic Appraisal
Economic Burden of Disease
Economic Competition
Economic costs
Economic Evaluation
Economic impact analysis
Economic Value of Life
Economic Value Theory
Economics
Employer Health Costs
Externalities
financial burden
Financial costs
Financial Management
Financing, Organized
Fixed costs
Friction cost method
Full Economic Evaluation
health care delivery
Health Care Financing
Health Care Market
Health Care Policy
Health Care Rationing
Health Care Reform
Health Care Sector
Health Care Utilization
Health Economics
Health Expenditures
Health Inequalities
Health Insurance
Health Maintenance Organization
Health Planning

Health Service Planning
Health Services Research
Health status index
Health status measure
Health Technology Assessment
Health years equivalent
Hospital Costs
Human capital approach
Incidence-base costs
Incidence-based approach
Incremental cost
Incremental cost-effectiveness ratio
Indemnity
Indirect benefits
Indirect costs
Industrial Organization
Insurance
Insurance Premiums
Intangible benefits
Intangible costs
Labor Economics
Managed Care
Marginal Analysis
Marginal Benefit
Marginal Cost
Markov model
Medicaid
Medical Practice Variation
Medicare
Methods of Benefit Assessment
Microcosting
National Health Expenditures
Opportunity Cost
Option Appraisal
Organization of Economic Cooperation and Development
Out-of-pocket Expenditures
Partial Evaluation
Pharmacoeconomics
Physician's Practice Patterns

Point of Service Plan
Preference weight
Preferred Provider Organization
Present values
Prevalence-based approach
Prevalence-based costs
Priority Setting and Rationing
Program costs
Programmatic cost analysis
Purchasing Power Parities
Quality adjusted life years
Regression method
regulatory impact analysis
Reimbursement
Remuneration Methods & Incentives
risk basis
Risk-based reimbursement
Self Insured Plan
Sensitivity Analysis
Shadow price
Social efficiency
Socioeconomic Determinants of Health
Socioeconomic Factors
Standard gamble approach
Technical Efficiency
Third party payer
Time preference
Underinsured
Uninsured
Utilization Review
Variable cost
Voluntary Care
Willingness to pay
