# Supplementary figures and images for: Analysis of the health economics portfolio funded by the National Institutes of Health in response to published guidance
Source: PLoS One. 2024 Feb 14;19(2):e0284235. doi: 10.1371/journal.pone.0284235 (PMC10866517; doi:10.1371/journal.pone.0284235)

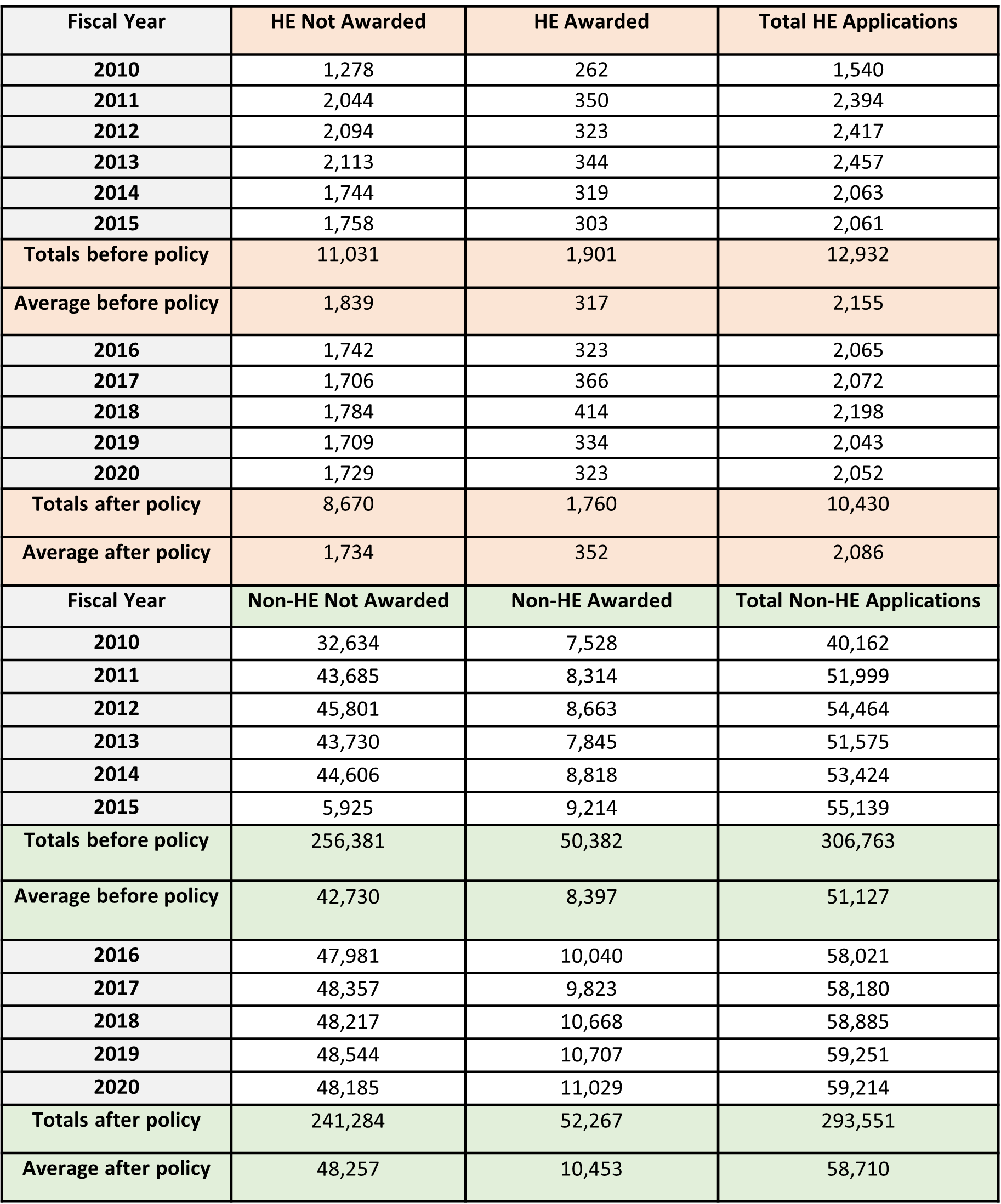

Supplement: S2 Table — Number of HE and non-HE projects before and following publication of the guidance. (TIF) [file pone.0284235.s002.tif]

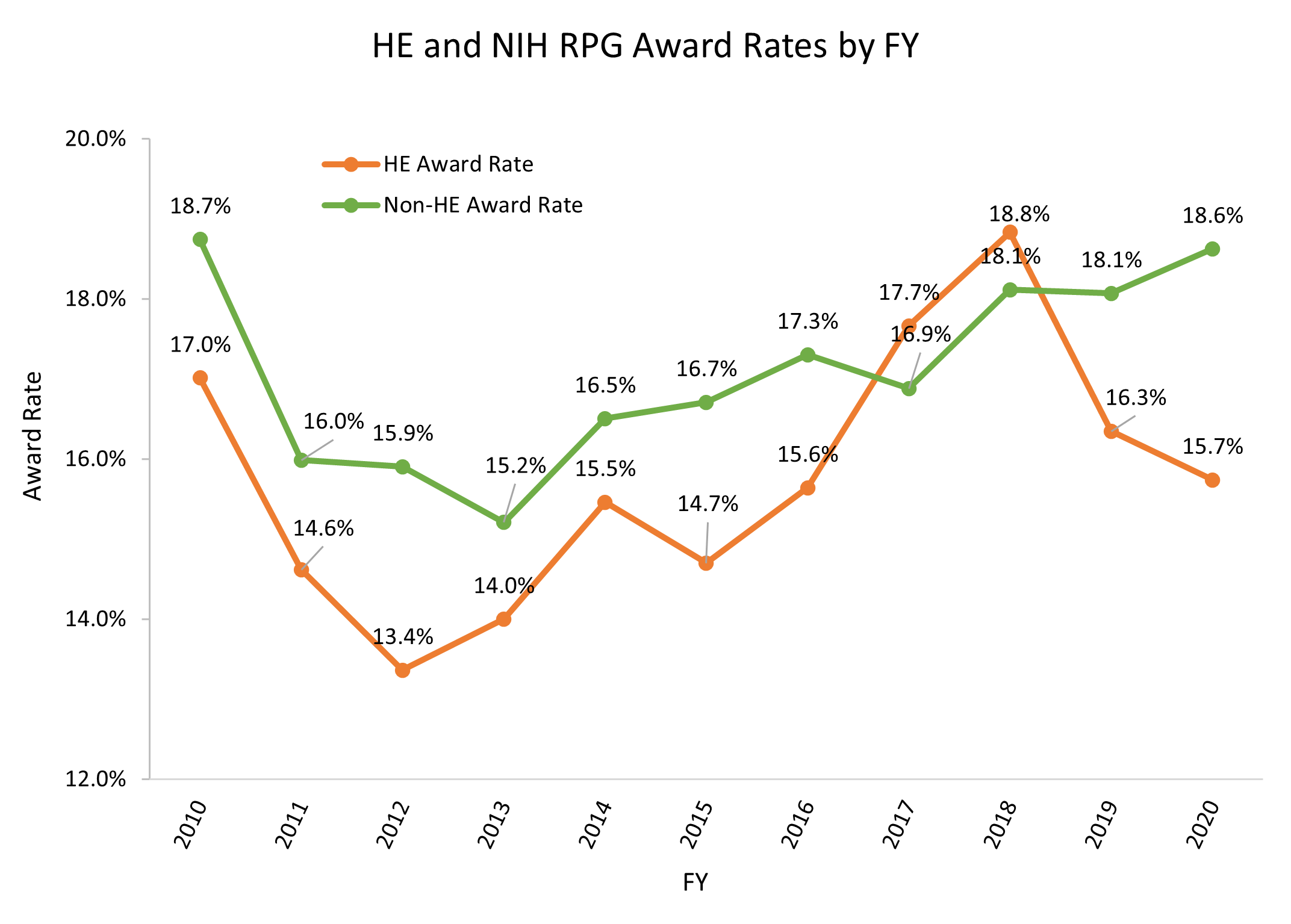

Supplement: S1 Fig — Award counts were divided by the total number of applications for HE and non-HE research for each Fiscal Year. (TIF) [file pone.0284235.s003.tif]

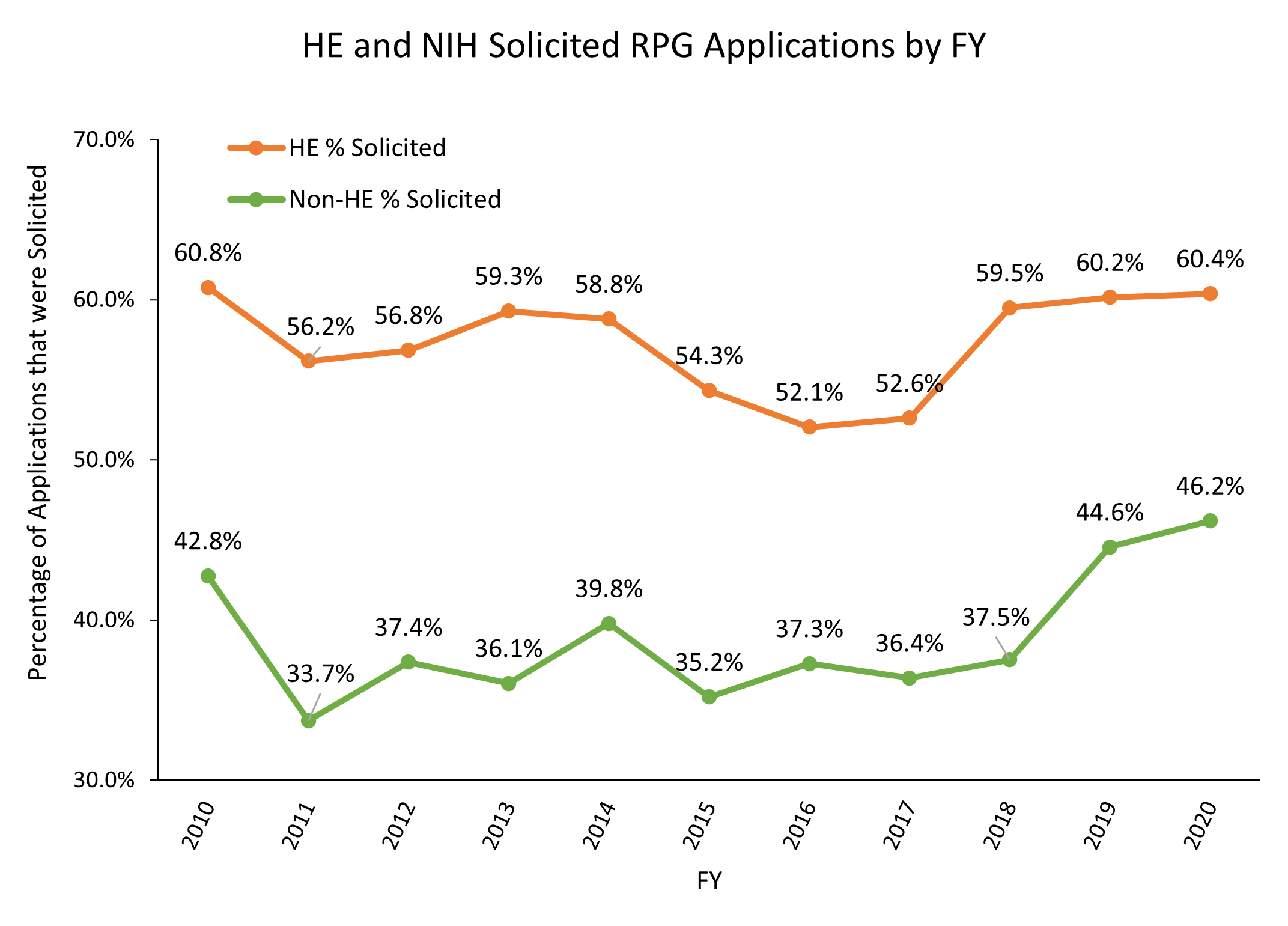

Supplement: S2 Fig — Application counts were divided by the total number of applications for HE and non-HE research for each Fiscal Year. (TIF) [file pone.0284235.s004.tif]

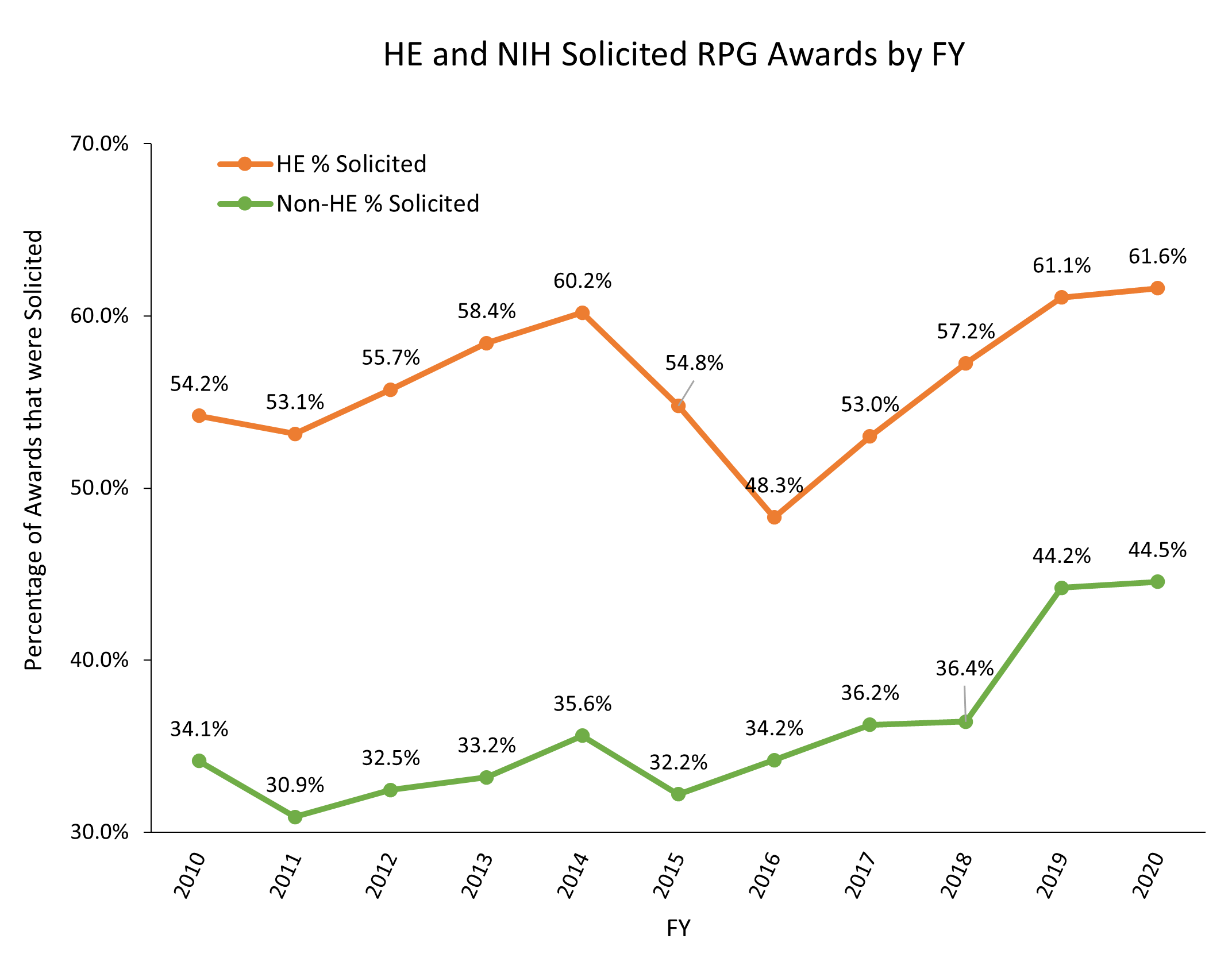

Supplement: S3 Fig — Award counts were divided by the total number of awards for HE and non-HE research for each Fiscal Year. (TIF) [file pone.0284235.s005.tif]

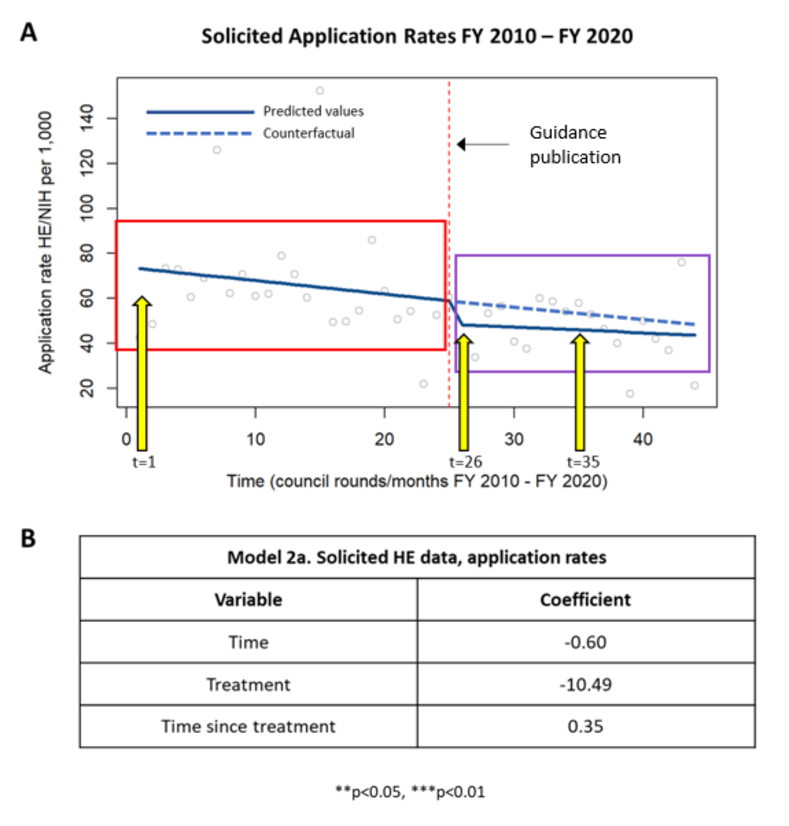

Supplement: S4 Fig — The red box outlines pre-policy time series points and the purple box, post-policy points. Arrows are pointing to time = 1, 26 and 35; explanations of their predictions are below. Coefficients are otherwise known as independent slopes (per variable) within the regression model. For the part of the graph outlined above in red, these are the pre-policy time points, therefore, with regard to the regression model, only the baseline constant and time variable are utilized in deriving this point (not intervention or time since intervention). For model 2a above, solicited application rates, the predicted outcome, can be derived using the necessary coefficients and values from the regression table, for example, at time = 1: Yt=β0+β11+β20+β30 Yt=73.90+−0.601+−10.490+0.350 Predicted Yt = 73.30 Looking at trends after the policy was put into place, outlined in the purple box on the graph above: Immediately after the policy change (May 2016), for this model (2a), time = 26, intervention = 1 and time since = 1: Yt=β0+β126+β21+β31 Yt=73.90+−0.6026+−10.491+0.351 Predicted Yt = 48.16 Looking further out (August 2018), post-policy change, for this model (2a), time = 35, intervention = 1 and time since = 10: Yt=β0+β135+β21+β310 Yt=73.90+−0.6035+−10.491+0.3510 Predicted Yt = 45.91 Had the policy not been put into place, a predicted value (counterfactual) can be calculated. This is visualized in the graph above with the dotted blue line continuing the same pre-policy trend/slope. For the same time = 35 example above: Yt=β0+β135+β20+β30 Yt=73.90+−0.6035+−10.490+0.350 Predicted Yt = 52.90 There is a slight difference in the slopes of the before and after the policy change. Therefore, at different time points, the predicted outcome will vary and may not always be higher had the policy not been put into place. (TIF) [file pone.0284235.s006.tif]

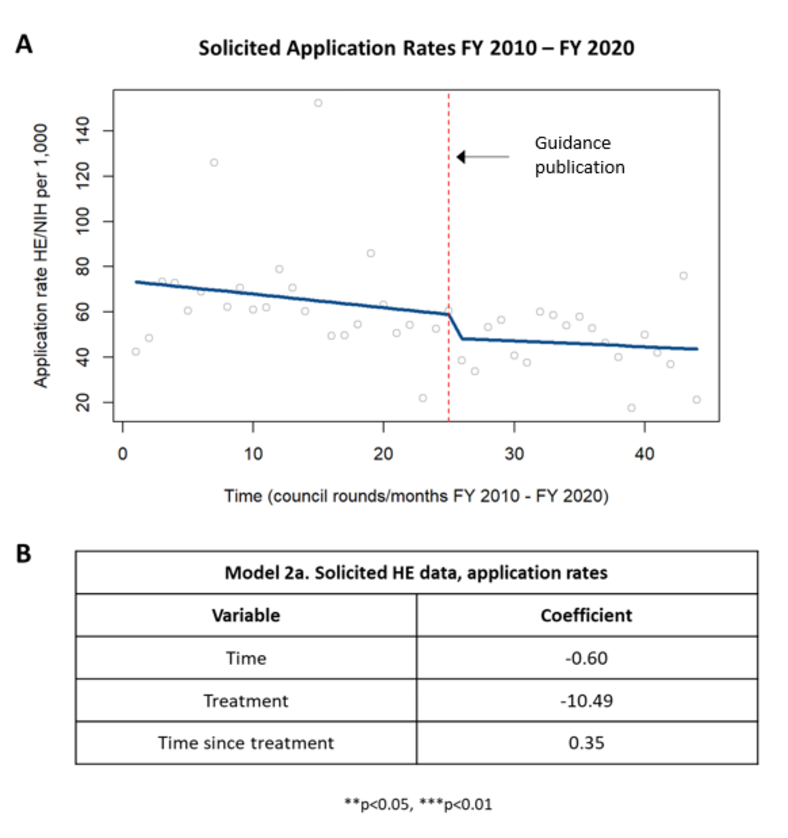

Supplement: S5 Fig — [A] Chart showing changes in application rates in solicited HE applications prior to and following the guidance publication in 2015. [B] Table summarizing the coefficients from the time series analysis. (TIF) [file pone.0284235.s007.tif]

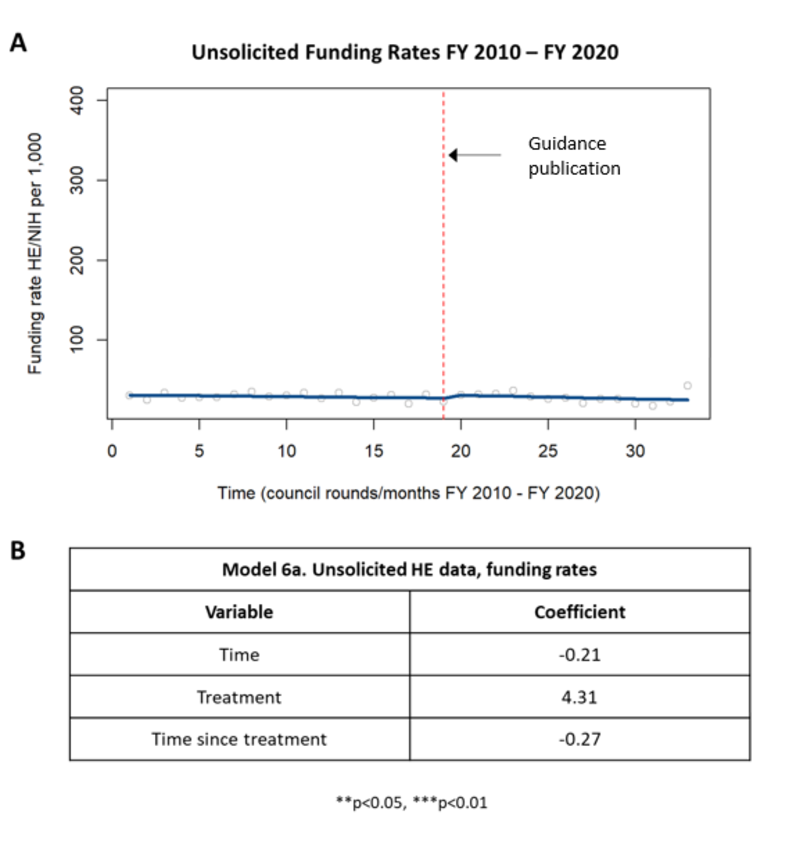

Supplement: S6 Fig — [A] Chart showing changes in funding rates in unsolicited HE applications prior to and following the guidance publication in 2015. [B] Table summarizing the coefficients from the time series analysis. (TIF) [file pone.0284235.s008.tif]

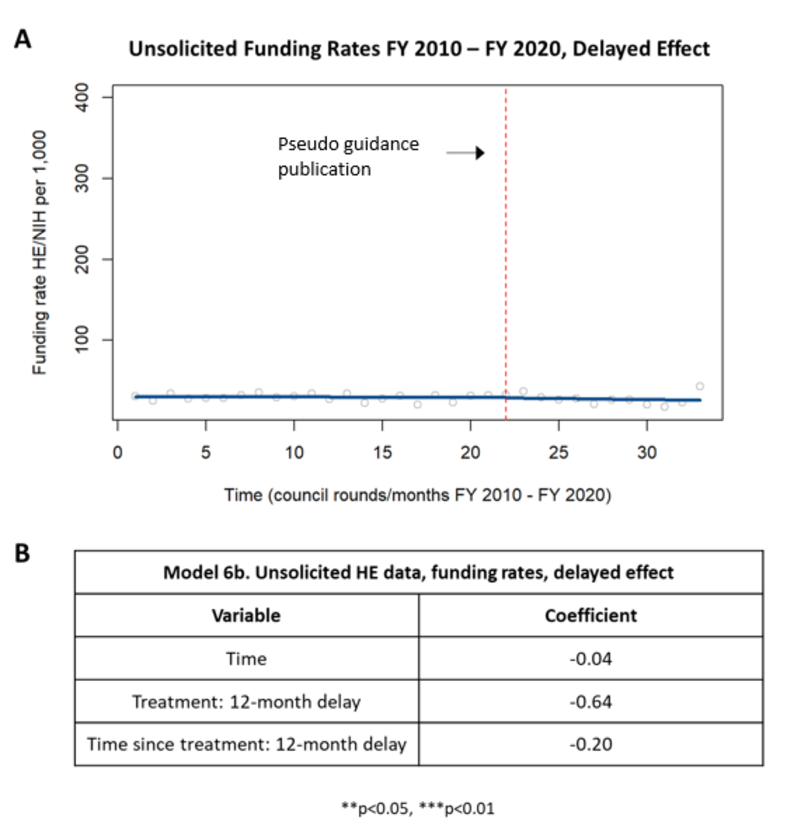

Supplement: S7 Fig — [A] Chart showing changes in funding rates in unsolicited HE applications up to one year after publication of the guidance and starting one year after publication of the guidance. [B] Table summarizing the coefficients from the time series analysis. (TIF) [file pone.0284235.s009.tif]
